# Spatial Variations in Seed Germination Traits of White Spruce (*Picea glauca*) and Black Spruce (*P. mariana*) Across the Canadian Boreal Forest

**DOI:** 10.3390/plants15060882

**Published:** 2026-03-12

**Authors:** Elaine Qualtiere, Yongsheng Wei, Dustin Snider, Yuguang Bai, Mark Johnston, Daniel W. McKenney, Pia Papadopol, Dale Simpson

**Affiliations:** 1 Bayer CropScience Inc., 17-410 Downey Road, Saskatoon, SK S7N 4N1, Canada; elaine.qualtiere@bayer.com; 2 Department of Plant Sciences, College of Life Sciences, Northwest A&F University, 22 Xinong Road, Yangling 712100, China; wysh70@126.com; 3 Department of Plant Sciences, College of Agriculture and Bioresources, University of Saskatchewan, 51 Campus Drive, Saskatoon, SK S7N 5A8, Canada; dustin.snider@usask.ca; 4 Saskatchewan Research Council, 125-15 Innovation Blvd, Saskatoon, SK S7N 2X8, Canada; johnstonm@sasktel.net; 5 Canadian Forest Service, Natural Resources Canada, 219 Queen Street East, Sault Ste. Marie, ON P6A 2E6, Canada; dan.mckenney@nrcan-rncan.gc.ca (D.W.M.); pia.papadopol@nrcan-rncan.gc.ca (P.P.); 6 National Tree Seed Centre, P.O. Box 4000, Fredericton, NB E3B 5P7, Canada; dale.simpson@nrcan-rncan.gc.ca

**Keywords:** *Picea glauca*, *Picea mariana*, seed viability, germination, base temperature, thermal time requirement, geographic variables, local adaptation, boreal forest regeneration, climate gradient

## Abstract

This study focuses on the spatial variation in seed germination characteristics of *Picea glauca* and *P. mariana*, prominent and widespread species within the Canadian boreal forest. The main objective was to determine seed germination requirements of geographically distinct seed collections of *P. glauca* and *P. mariana*. A total of 73 collections of *P. glauca* and 62 collections of *P. mariana* were selected across Canada and tested for germination under various temperatures. Base temperature (*T_b_*) and thermal time required to reach 50% germination (*TH_50_*) were derived from thermal model parameters for all seed collections. Correlation analyses between seed germination traits, geographic, and climatic variables were conducted. Base temperatures for germination of *P. glauca* ranged from 5.2 to 11.9 °C while *P. mariana* had base temperatures ranging from 6.2 to 12.8 °C, indicating a broader temperature range for the former to initiate germination. Optimal germination temperatures ranged from 15 to 20 °C for *P. glauca* and from 17.5 to 30 °C for *P. mariana*. Thermal time requirements for 50% germination were higher for *P. glauca* than for *P. mariana,* indicating that the former takes longer to germinate under the same temperature conditions. Latitudinal-related variables such as temperature of sites had a stronger influence on germination relative to precipitation or potential evaporation and affected seed viability, final germination and germination capacity of all seed sources. Seed viability was lower in northern seed collections and germination capacity was diminished at lower temperatures for both species. The results from this study can be built into models predicting shifts in boreal forest species under climate change.

## 1. Introduction

Plant population dynamics in disturbance-driven boreal forest systems are strongly influenced by natural regeneration. This process consists of many life cycle stages such as dispersal, predation, germination and establishment [1]. The success and timing of germination of plant species influence the overall success of natural regeneration. Therefore, the ability to predict germination events across large spatial regions will lead to an improvement in our understanding of regeneration dynamics within the Canadian boreal forest.

The Canadian boreal forest is composed of a mixture of cool coniferous and deciduous tree species [2]. *Picea mariana* (Mill.) BSP and *Picea glauca* (Moench) Voss are widespread and often dominant climax species in their respective sites within this region [3]. *P. glauca* is adapted to a wide range of soil and climatic conditions compared to many other species but is still somewhat site-demanding and often restricted to sites with well-drained, basic mineral soils [4]. It grows poorly on sites with high water tables and is intolerant of permafrost [5,6]. Conversely, *P. mariana* usually grows on wet organic soils, with productive stands found on a variety of soil types [7]. These species have a wide distribution, extending from Newfoundland to Alaska and into the Northern United States. *P. glauca* is one of the northernmost tree species in North America, reaching north of 69° N latitude in the Mackenzie River delta [8]. *Picea mariana* has a similar distribution to *P. glauca*; however, it grows under a larger range of site conditions such as wet organic soils (bogs or fens) as well as upland regions [9].

Spatial variation in plant life history and ecological traits is common [10]. Seed traits, such as seed mass or size, seed production, germination, and dormancy can vary along latitudinal, longitudinal and altitudinal gradients. For example, seed mass or size generally decline with latitude [10,11,12,13,14,15]. Seed mass increases longitudinally from the temperate east coast to the arid interior for *Glycine* species across Australia [16]. Altitude is negatively correlated with seed mass for *Pedicularis* species in the eastern Tibetan Plateau [17]. Seed-setting of *Calluna vulgaris* (L.) Hull generally declined with increasing site ocean proximity and altitude in Scotland [18], and seeds of *C. vulgaris* and *Erica cinerea* L. collected at the highest altitudes reached highest final germination [19]. However, latitude and altitude influenced reproductive traits of *Anthyllis vulneraria* L. differently, with seed set and seed mass decreasing with latitude but showing no response to altitude [20]. Seed dormancy and germination traits in *Koenigia islandica* L. vary along a latitudinal gradient, influenced by mean summer and winter temperatures, with conditional dormancy and scarification requirements more pronounced under cooler summer temperatures [21].

Differences in seedling establishment along altitudinal, latitudinal, and longitudinal gradients also appear to be species-specific. The seedling establishment of *Pseudotsuga menziesii* (Mirb.) Franco in the southern Rocky Mountains of Colorado is limited by soil moisture at high elevations [22]. *Pinus flexilis* E. James in Colorado shows a similar elevation-related constraint, where seasonally low soil moisture limits establishment at higher elevations [23]. In contrast, *Pinus douglasiana* Martínez and *Pinus maximinoi* H.E.Moore are restricted at higher elevations due to thermal requirements (T_b_ and *θ_50_*) [24].

Germination modelling has been developed extensively within the agronomic field. However, knowledge of temperature thresholds, such as base temperatures, thermal time requirements, and quantitative effects of temperature on germination, is lacking for tree species. Thermal time models offer a large degree of utility because model parameters are physiologically significant [25,26]. Studying spatial variation in germination requirements, variations between species and populations, and adaptations to local climatic conditions is critical for understanding the ecological adaptation of boreal tree species and their response to climate. The base temperature for germination is the minimum temperature at which germination can occur and thermal time is the heat accumulation above a base temperature [27]. Many studies have illustrated the effectiveness of using thermal time models to predict germination timing in a variety of species [28,29,30,31,32].

Identifying spatial patterns in germination thresholds can help us better understand environmental constraints on the distribution and dynamics of boreal forests, supporting the development of effective conservation, adaptation, and reforestation strategies. This study focuses on the Canadian boreal forest and the spatial variation in seed germination characteristics of *P. glauca* and *P. mariana*, prominent and widespread species within this biome. The objectives were to (1) determine thermal time parameters for germination of various collections of *P. glauca* and *P. mariana* across the Canadian boreal forest; (2) identify climatic variables controlling seed germination response to temperature in these species; (3) identify spatial patterns in seed germination in these species; and (4) interpret species and population responses to temperature across environmental gradients. It was hypothesized that (1) the requirements for germination, such as base temperature (*T_b_*) and thermal time requirements (*TH_50_*), vary among species and collections; and (2) germination traits vary along latitude and longitude gradients, both across the Canadian boreal forest and within specific ecozones. The majority (72%) of the collections were located within the Boreal Plains and Boreal Shield ecozones; therefore, analyzing seed sources based on ecozone location may clarify important climatic factors related to seed germination.

## 2. Results

### 2.1. Plasticity in Temperature Requirement During Germination and Spatial Variation in Viability and Germination

#### 2.1.1. Seed Viability and Germinability

**Figure 1:**
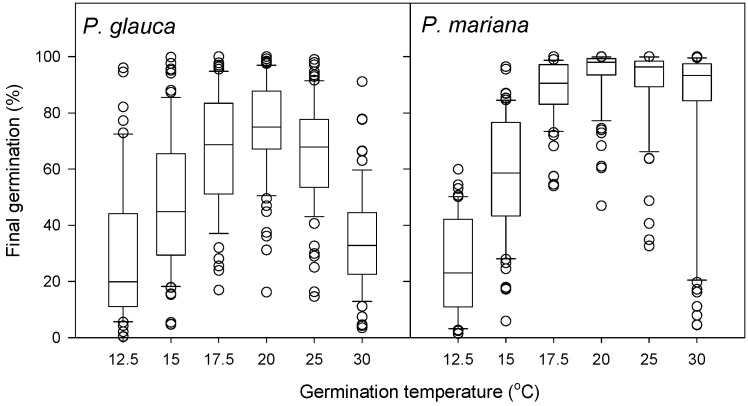
Final germination percentage (%) for *Picea glauca* and *P. mariana* seeds collected from across the Canadian boreal forest at temperature regimes between 12.5 and 30 °C.

Seed viability was 89% (n = 73) in *Picea glauca* and 94% in *P. mariana*. There was no significant difference in seed viability between Boreal Shield and Boreal Plains ecozones within each species. Final germination percentage of viable seeds was high for both species (Figure 1). The majority of *P. mariana* collections had especially high germination (>80%) while *P. glauca* collections demonstrated variable germination across the temperature regime.

The optimal temperature for germination, based on the highest observed final germination, was 20 °C for most *P. glauca* collections, ranging from 15 to 25 °C. Germination was reduced at 30 °C in all *P. glauca* collections, showing that this species has limited adaptation for germination under high temperatures. The optimal temperature range for germination was higher and broader in *P. mariana* with more consistent germination rates among collections within the range of 17.5 to 30 °C.

**Figure 2:**
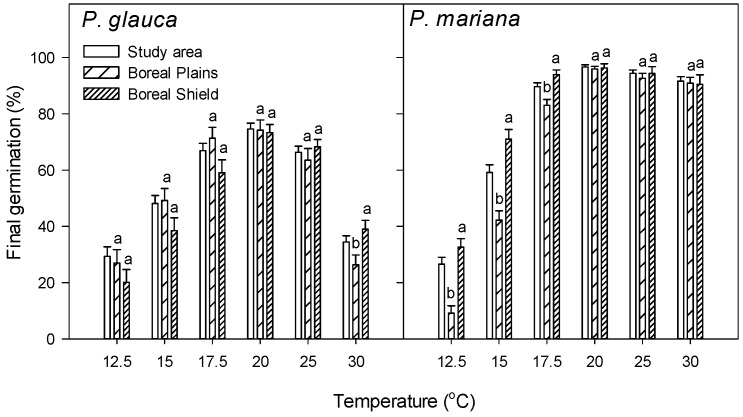
Final germination percentage (%) of *Picea glauca* and *P. mariana* collected from the Canadian boreal forest and within two selected ecozones at temperature regimes between 12.5 and 30 °C The error bar is SE. The small letter represents significant difference at *p* ≤ 0.05 between the Boreal Plains and Boreal Shield at same germination temperature within species.

There were no significant differences in final germination of *P. glauca* between the Boreal Plains and Boreal Shield ecozones at temperatures between 12.5 and 25 °C, but it was significantly higher in Boreal Shield at 30 °C (Figure 2). However, final germination of *P. mariana* in Boreal Shield was significantly higher than that in Boreal Plains in the temperature range from 12.5 to 17.5 °C, and there was no significant difference between them at 20 to 30 °C.

#### 2.1.2. Correlations Between Climatic Variables and Seed Viability and Final Germination

*Picea glauca* seed viability had strong, positive correlations (r = 0.40 to 0.50) with annual and summer potential evaporation (PE) in the Boreal Plains (Table 1). *P. glauca* seed vitality had negative correlations with end of growing season and length of growing season in the Boreal Plains (r = −0.41 and −0.40, respectively). The only correlation between temperature variables and seed viability for *P. glauca* in the Boreal Plains was with minimum April temperature (r = −0.40).

*Picea mariana* seed viability displayed positive correlations with annual potential evaporation (r = 0.30) and annual mean and maximum temperatures (r = 0.26 and r = 0.34, respectively) across the boreal forest (Table 1). The same patterns occurred within the Boreal Shield ecozone (r = 0.53, r = 0.40 and r = 0.51, respectively). In the Boreal Shield, a negative correlation occurred between seed viability and the Julian date of the start of growing season (r = −0.42). Interestingly, seed viability was not correlated with precipitation for either species, possibly due to the high seed viability of the two species.

Final germination percentage of *P. glauca* at germination temperatures from 15 to 25 °C had positive correlations with annual and summer PE and negative correlations with annual and winter precipitation in the Boreal Plains ). However, these relationships were reversed in the Boreal Shield. Final germination percentage of *P. glauca* at temperatures 12.5 to 20 °C had negative correlations with temperature annual range, temperature seasonality and summer mean temperatures, as well as positive correlations with winter mean temperatures in the Boreal Shield. In the Boreal Plains, final germination percentage had significant positive correlations only with summer mean temperatures.

For *P. mariana*, few correlations were found between final germination percentage and climatic variables in the Boreal Plains. However, negative correlations between final germination at 12.5 to 15 °C were found for annual and summer PE and summer mean temperatures in the Boreal Shield. Final germination at 12.5 to 17.5 °C had positive correlations with precipitation in the Boreal Shield. These relationships suggest that germinability of *P. mariana* seeds at lower germination temperatures (12.5 to 15 °C) was reduced by dry and warm conditions in the Boreal Shield.

#### 2.1.3. Correlations Between Geographic Variables, Seed Viability, and Final Germination Percentage

Seed viability of *P. glauca* increased from west to east and increased from north to south across the boreal forest (Table 2). Final germination percentage of *P. glauca* at 30 °C was positively correlated with longitude (r = 0.35) and negatively correlated with latitude (r = −0.31) across the boreal forest (Table 2). Positive correlations between longitude (from west to east) and final germination percentage were found at all germination temperatures within the Boreal Shield (r = 0.45 to 0.79). Final germination of *P. glauca* at most germination temperatures had a positive correlation with the Julian day of the end of growing season within the Boreal Shield. Across the boreal forest, it was positively correlated with the Julian day of the start of growing season.

The final germination percentage of *P. mariana* at 12.5 to 17.5 °C increased from west to east across the boreal forest and the Boreal Shield (Table 2). Negative correlations between final germination percentage at 12.5 to 20 °C and latitude were found across the boreal forest but not within any ecozone. Final germination percentages at higher temperatures (25 to 30 °C) increased from north to south in the Boreal Shield. Final germination of *P. mariana* at 15 °C had negative correlations with the Julian day of the end and the length of the growing season within Boreal Plains. Final germination at 12.5 and 15 °C had a positive correlation with the Julian day of the start of the growing season, and that at 12.5 to 17.5 °C had a positive correlation with the Julian day of the end of the growing season.

### 2.2. T_b_ and TH_50_ as Adaptive Traits and Spatial Variability

**Figure 3:**
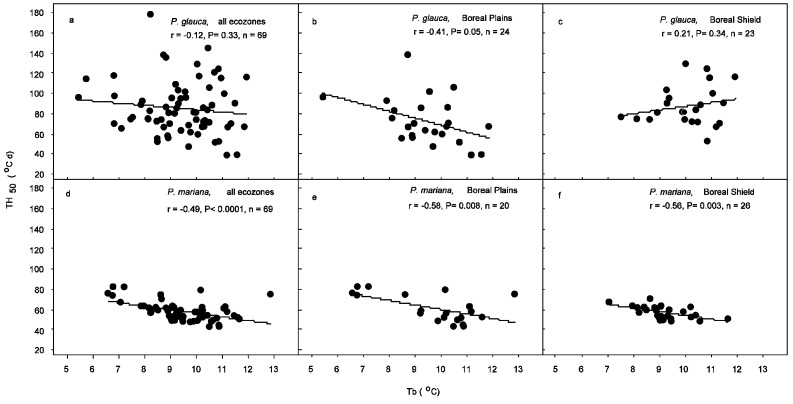
Relations between base temperature (*T_b_*) and thermal time requirement for 50% germination (*TH_50_*) in *Picea glauca* (**a**–**c**) and *P. mariana* (**d**–**f**) across the Canadian boreal forest (**a**,**d**) and within the Boreal Plains (**b**,**e**) and the Boreal Shield (**c**,**f**) ecozones.

The range of base temperature for germination (*T_b_*) was 5.2 to 11.9 °C for *P. glauca* and 6.2 to 12.8 for *P. mariana* across the boreal forest. There was no significant difference between the two species. There was no significant difference in *T_b_* between Boreal Plains and Boreal Shield ecozones for either species. Thermal time required to reach 50% germination (*TH_50_*) was greater for *P. glauca* (85.2 ± 3.2 °C·d) than *P. mariana* (58.2 ± 1.2 °C·d). *TH_50_* was lower in Boreal Plains (72.4 ± 4.7 °C·d) than Boreal Shield (87.5 ± 4.1 °C·d) for *P. glauca*, while it was not significantly different between the two ecozones for *P. mariana*. T_b_ of *P. glauca* had a negative correlation with *TH_50_* only within the Boreal Plains ecozone, while that of *P. mariana* was negatively correlated with *TH_50_* across the boreal forest and within both ecozones (Figure 3).

There was no significant trend in *T_b_* along the gradients of longitude and latitude across the entire boreal forest for the two conifer species (Table 3). However, *T_b_* decreased with longitude (from west to east) in *P. glauca* within the Boreal Shield and increased in *P. mariana* within the Boreal Plains. *T_b_* of *P. mariana* decreased with increasing latitude within the Boreal Plains. *TH_50_* of *P. glauca* had a negative correlation with latitude across the boreal forest and a strong negative correlation with longitude within the Boreal Shield. No other correlations were significant with regard to TH_50_.

*T_b_* had no correlations with any climatic variables within the Boreal Plains for *P. glauca* or within the Boreal Shield for *P. mariana*. Across the boreal forest, *T_b_* of *P. glauca* only had a positive correlation with temperature annual range (the difference between the maximum temperature of the warmest period and the minimum temperature of the coldest period) (Table 4). Within the Boreal Shield, strong correlations were found between *T_b_* and most climatic variables for *P. glauca.* *T_b_* increased with increasing annual potential evaporation, precipitation seasonality, temperature seasonality, temperature annual range and growing season temperatures, and decreased with the Julian date of the end of the growing season, precipitation and winter temperature. A similar trend was found for *P. mariana* collections, but most coefficients were not significant.

No correlations between *TH_50_* and climatic variables were found in *P. mariana*. *P. glauca*, however, displayed more correlations between *TH_50_* and climatic variables across the boreal forest than within any ecozone (Table 5). *TH_50_* increased with increasing growing season length, potential evaporation, and temperature, but its relationship with precipitation was inconsistent.

## 3. Discussion

### 3.1. Seed Viability

The distribution of forest zones in the boreal biome is controlled by the production of viable seeds [33,34]. Assumptions based on previous studies were that seed viability would decrease in more northern collections, with greater germination found in the Boreal Plains relative to the Boreal Shield in the boreal forest [15,35,36,37]. Our results showed that in general, viability of *P. mariana* declined from south to north, but that of *P. glauca* declined from southeast to northwest. Other studies found similar patterns in *P. mariana* seeds, which exhibited lower seed viability at high latitudes due to lower heat sums [34,35,38]. Seed viability of *Alnus viridis* ssp. *fruticosa* was also higher in the southern relative to northern areas of the Mackenzie Delta region, Northwest Territories, Canada [36]. *Juniperus communis* seed viability strongly declines towards regions having harsher environments, such as long, colder winters [39]. Low temperatures result in low seed viability in high-latitude regions due to maladaptation of seed characteristics [36]. There is repeated evidence that production of viable seeds is limited in populations at their northern limits [40,41,42,43,44] as colder environments negatively influence seed development in gymnosperms by diminishing pollination success and increasing ovule abortion and embryo losses after fertilization [34,45,46,47,48,49,50]. Therefore, the reduction in seed viability in northern locations is likely due to a multitude of factors brought on by colder environments, which negatively influence seed development.

Positive correlation between seed viability of *P. mariana* and the length of growing season within the Boreal Shield indicates that increased growing degree days benefit seed viability of this species. This reinforces the above findings that colder temperatures had a negative effect on seed viability and short growing season may be the limiting factor for seed development and germination in this region. Embryos of *P. mariana* required 800–940 degree days to reach 100% maturity in the northern boreal forest [51]. *P. glauca* had a contrasting pattern within the Boreal Plains, with seed viability decreasing with increasing length of growing season. The Boreal Plains has lower precipitation and warmer temperatures relative to the Boreal Shield; therefore, lack of moisture or increasing heat may become an issue during longer growing seasons and may impede seed development in this species. On the other hand, no correlation between viability and precipitation was found in either species, suggesting that seed viability is a function of temperature more than precipitation. For forests in the northern hemisphere, low temperatures often limit the viability of plant populations at their northern boundary [42,52,53,54,55,56], while water availability is the main limiting factor at the southern boundary [57,58,59,60].

### 3.2. Seed Germination

Final germination was high in both species but varied among species and ecozones as we predicted. Optimum germination temperatures for *P. mariana* were 17.5 to 30 °C, which were wider and higher than those of *P. glauca* (17.5 to 25 °C), indicating that *P. mariana* is better adapted to warmer temperatures and has a wider range of temperatures for germination. Overall, *P. mariana* seed collections from more northern locations germinated optimally at lower temperatures compared to those from southern locations, demonstrating the importance of matching seed sources to local thermal conditions.

Across the boreal forest, final germination of *P. glauca* was negatively correlated with latitude and positively correlated with longitude only when germinated at 30 °C (Table 2), while *P. mariana* had correlations with latitude and longitude at 12.5, 15 and 17.5 °C. This suggests that *P. mariana* has a stronger latitude and longitude germination pattern than *P. glauca*. Both *P. glauca* and *P. mariana* had positive correlations with longitude in the Boreal Shield. A previous study found that seed germinability decreased from 53° N to 58° N in Quebec-Labrador [61], which displayed a similar latitudinal pattern in *P. mariana* as in our study.

*Picea glauca* seed sources are highly adapted to their provenances and this is exhibited by its variable germination among locations. In the Boreal Shield, final germination of *P. glauca* increased with increasing precipitation. Seeds of *P. glauca* from relatively wetter and colder sites had greater germinability than those from drier and warmer sites. However, in the Boreal Plains, the interaction between germination and precipitation was contrary to the Boreal Shield. In the Boreal Plains, final germination decreased with increasing precipitation. These differing results in germination and precipitation interactions show the plasticity of *P. glauca* through its ability to adapt to its local environments: in wetter regions it has increased germination under higher precipitation and the opposite is true in drier regions.

Negative correlations between final germination at 12.5 to 15 °C, growing season potential evaporation, and summer temperature were found in *P. mariana*. There were positive correlations between final germination and precipitation in the Boreal Shield. Thus, *P. mariana* seeds collected from wetter and colder sites can reach higher germination percentage at lower temperatures (12.5 to 15 °C). These patterns have been observed in *P. mariana*, where temperature explained the 85% variation in germination percentage among sites [61].

### 3.3. Correlation of Germination Traits with Climatic and Geographic Variables

The average base temperature for germination (*T_b_*) of the two species was about 9.5 °C, which is several degrees higher than grassland species to the south of the boreal forest [30,31], possibly a mechanism to prevent premature germination when temperature is low. It is higher than the *T_b_* (5 °C) frequently used for calculating thermal sum in boreal forest [61]. Both *T_b_* and thermal time requirement for 50% germination (*TH_50_*) varied among ecozones and species. *T_b_* of *P. mariana* was slightly higher than that of *P. glauca*, indicating that *P. mariana* requires warmer temperature to initiate germination. However, there were no significant differences in T_b_ along gradients of longitude and latitude across the boreal forest for either species. This suggests that *T_b_* is a relatively stable trait in the two species at the continental scale. However, on the scale of ecozones, *T_b_* increases from east to west in *P. glauca* within Boreal Shield. In the Boreal Plains, *T_b_* of *P. mariana* decreases from east to west and with increasing latitude (Table 3). These results indicate that wetter regions were associated with a lower *T_b_* for *P. glauca* within Boreal Shield, but a higher *T_b_* for *P. mariana* within Boreal Plains. These results suggest that *P. mariana* starts germination later in wetter environments. A strong negative correlation between *T_b_* of *P. glauca* and precipitation (annual and monthly from August to May) within Boreal Shield provides strong evidence for this conclusion (Table 4). A similar relationship has been documented in *Festuca hallii,* where sites with greater precipitation and lower temperature had heavier seeds, which usually had lower thermal time requirements for germination and faster germination [62]. Larger seeds can germinate faster under stressful conditions and longitudinal patterns in seed germination were correlated with precipitation [62].

*T_b_* of *P. glauca* also had a positive correlation with mean temperature of warm season (from May to August), potential evaporation (from May to August) and negative correlation with mean temperature of cool season (from November to February) within Boreal Shield (Table 4). Positive correlations between *T_b_* with temperature variables were also found in *P. mariana* within the Boreal Plains. At the scale of the Canadian boreal forest, T_b_ of *P. glauca* had a positive correlation with annual temperature range (highest mean monthly temperature—lowest mean monthly temperature), while T_b_ of *P. mariana* increased with the potential evaporation from July to September.

*TH_50_* of *P. mariana* (58.2 ± 1.2 °C·d) was significantly lower than *P. glauca* (85.2 ± 3.2 °C·d) across the boreal forest, which shows that *P. mariana* seed collections germinated faster than *P. glauca* seeds. As a semi-serotinous spruce species, *P. mariana* often has a shorter germination period, which is thought to be necessary for achieving seedling growth in cooler conditions [63,64]. No correlations were found between *TH_50_* of *P. mariana* and climatic or geographic variables. However, *TH_50_* of *P. glauca* had a negative correlation with latitude across the boreal forest and with longitude within the Boreal Shield. Therefore, germination time decreased in more northerly collections across the entire boreal forest and from west to east within the Boreal Shield. These latitude and longitude patterns were further demonstrated by positive correlations with temperature-related factors (mean, max, and min temperatures) and negative correlations with CV of precipitation across the boreal forest. Overall, seeds from cooler regions required less thermal time to reach 50% germination.

We found negative correlations between *T_b_* and *TH_50_* for *P. glauca* within the boreal forest, the Boreal Plains, and Boreal Shield, and for *P. mariana* within the Boreal Plains. This suggests that species and populations with a higher *T_b_* may require less thermal time for germination to ensure successful seedling recruitment as reported in *Festuca hallii* [62].

While we focused on thermal requirements regulating seed germination in *Picea glauca* and *Picea mariana*, there are recent developments that provide additional tools for understanding and predicting the current distribution and future responses of boreal tree species to climate change. Species distribution modelling (SDM) is now widely used to predict a species’ range under current and future environmental conditions [65,66]. Numerous studies have used SDM to predict both present and future distributions of *Picea* [9,66,67,68]. SDM projections indicate that suitable habitats for both *P. glauca* and *P. mariana* are likely to contract within the next few decades under IPCC RCP climate scenarios [9,69]. However, these models often do not account for early life-history processes that can limit the success and rate of regeneration. These modelling techniques have been combined with germination experiments for other species, including research on threatened *Saussurea* spp. that jointly modelled climatic suitability in southwest China [70]. As SDM approaches continue to develop, incorporating germination thresholds could improve predictions of future ranges for *Picea* spp. under future climate conditions.

A limitation of these methods is the uncertainty of future climates and disturbance regimes. Future fire patterns cannot be accurately predicted, and yet they play a substantial role in the distribution and range dynamics of boreal trees. Shorter fire intervals (less than 25 years) can reduce *P. mariana* recruitment by burning stands before trees reach reproductive age, removing seed sources and preventing post-fire regeneration [71,72]. Trembling aspen (*Populus tremuloides*) has improved recruitment under more frequent fires through suckering, leading to regeneration of stands that are less conifer-dominated [73].

## 4. Materials and Methods

### 4.1. Seed Sources

**Figure 4:**
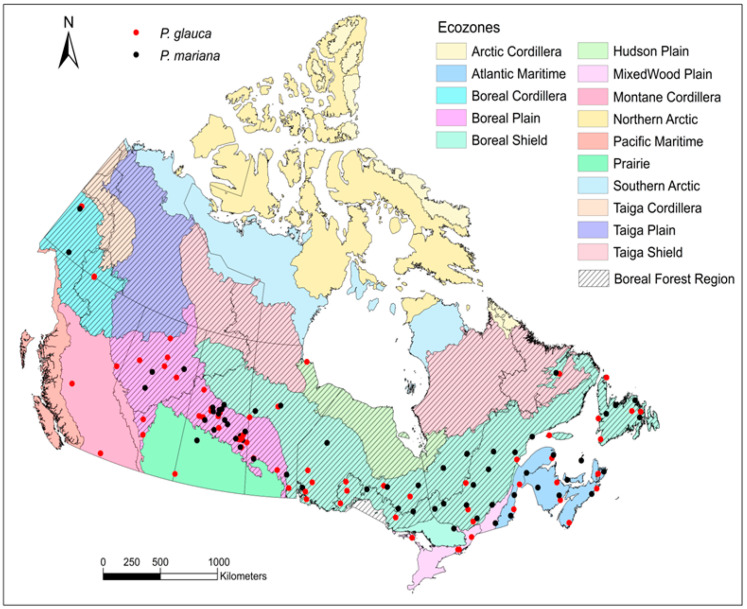
Location of seed collections of *Picea glauca* and *P. mariana* across Canada. Note that several collections are from outside the boreal forest distribution range.

*Picea glauca* and *P. mariana* seed collections were obtained from the Weyerhaeuser Seed Orchard, north of Prince Albert, Saskatchewan, and the National Tree Seed Centre (NTSC), Canadian Forest Service, Fredericton, New Brunswick. The selection of seed collections from the Weyerhaeuser Seed Orchard was based on availability. Seeds from the National Seed Centre were based on the availability of recent collections with high seed germination percentage and low elevations covering a wide geographic range. A total of 73 collections of *P. glauca* and 62 collections of *P. mariana* were selected across the forested regions of Canada; one exception included a single collection from the Prairies. Nine terrestrial ecozones were covered by these collections within a range of 43.77° N to 63.38° N and from −136.42° W to −53.90° W (Figure 4, Table S1). Seeds were stored at −20 °C for 1–10 years after collection until used in germination experiments.

Tetrazolium (TZ) tests were conducted to assess seed viability following procedures established by the International Seed Testing Association [74] and Grabe [75]. Five replicates of 10 seeds from each collection were soaked in water and 0.1% 2, 3, 5- Triphenyl tetrazolium chloride solution for 24 h in darkness. Seeds were then dissected under a microscope and those that remained unstained or mottled in colour were considered non-viable.

### 4.2. Thermal Time Modelling: Quantifying Thermal Requirements for Germination

Germination experiments were conducted in growth chambers (MLR-350H, Sanyo Scientific, New York, NY, USA) with constant-temperature regimes at 12.5, 15, 17.5, 20, 25 and 30 °C with 12/12 h light/darkness. Temperatures inside the growth chambers were monitored continuously using dataloggers (21× Campbell Scientific Inc., Logan, UT, USA) and recorded at hourly intervals using three temperature probes per chamber. Measured temperatures were used for model development, which were 12.6, 14.9, 17.3, 20.1, 25.2 and 29.8 °C, respectively.

A randomized complete block design (RCBD) with five replications was used; replicates were put into growth chambers at one-week intervals. For each replicate, 25 to 60 seeds, depending on seed availability, were imbibed on top of two layers of filter paper (Whatman ^®^ No. 1) moistened with 5 mL of distilled water in 9 cm plastic Petri dishes. Petri dishes were enclosed in clear plastic bags to reduce evaporation and randomized within each growth chamber at each temperature. During the course of the experiments, seeds with any sign of fungal infection were sprayed with 95% ethanol solution (Wang et al., 2003) [30]. Distilled water was added periodically when needed. Germinated seeds were counted and removed every 24 h. Seeds were considered germinated when the radicle was ≥2 mm in length.

Cumulative germination percentage was calculated for each species and temperature treatment. Cumulative germination for each seed collection was transformed to a scale of 0–100% by dividing cumulative germination percentages by a scaling factor based on the percentage of viable seeds [26,29]. A Chapman–Richards function, $y=a{\left(1-e^{-bx}\right)}^{c}$ [76], commonly used to describe cumulative growth curves, was used to model germination time courses in SigmaPlot 10.0 (Systat Software Inc., San Jose, CA, USA, 2006). In this equation, *y* is accumulated germination over time, *x* represents the maximum accumulated germination and parameters *b* and *c* control the curve shape. Seed populations were considered to be composed of subpopulations (10, 20, 30, 40, 50, 60, 70, 80, and 90%), which were assumed to germinate in the same relative order regardless of thermal environment [28,29]. The number of days required to achieve 10–90% germination was calculated from the modelled germination time course based on the Chapman–Richards function. A non-linear regression procedure in SAS (Version 6.11, Proc NLIN) was used to estimate germination time (*t_g_*) for the subpopulations [31]. Germination rate for subpopulation g (GR_(g)_) was calculated with the reciprocal of germination time (1/*t_g_*):

$${GR}_{\left(g\right)}=\frac{1}{t_{g}}=\frac{T-T_{b}}{\theta_{T(g)}}$$

where *GR_(g)_* is considered to be linearly related to temperature (*T*) within the suboptimal range between base temperature (*T_b_*) and optimal temperature (*T_o_*) for non-dormant seeds [28]. The range of suboptimal temperatures was established based on visual inspection of the linear relation between germination rate and temperature [30,31]. Germination temperature was plotted against *GR_(g)_* and values that deviated from a linear relationship were excluded from the calculations and were considered to be supra-optimal temperatures. The intercept of the linear regression line was estimated using graphical extrapolation techniques [28] and was considered the T_b_; a single T_b_ was assumed for all subpopulations. If the variation in *θ_T(g)_* within a seed population followed a normal distribution, the germination time course in terms of thermal time could be described by a Probit equation [26,30]. The linear relationship between temperature (*T*) and *GR_(g)_* varied among subpopulations as indicated by the slope of the regression line, which equals the thermal time requirement of subpopulation (*TH_(g)_*). The time to reach 50% germination was calculated using the Chapman 3-parameter function for each collection with a final germination over 50% [31]. Probit analysis was then used to estimate θ_T(50)_ and σθ_T_ (standard deviation of thermal time) for each seed collection, and was used in the application of the thermal time model.

### 4.3. Climatic and Geographic Variables

Baseline climatic data (1971–2000) for the study area were obtained from a national historical climate dataset [77,78,79]. This gridded data uses an ANUSPLIN model, which is a multivariate non-parametric surface-fitting approach to develop a spatially continuous climate model or “surface” [80]. This is especially important for forest applications because weather station data is rarely available in remote locations. A total of 72 climatic variables and two geographic variables (latitude and longitude) were generated for the coordinate locations associated for each of the seed populations.

### 4.4. Correlations Between Seed Germination Parameters and Environmental Variables

Base temperature (*T_b_*) and thermal time requirement for 50% germination (*TH_50_*) were checked for potential outliers using the Cleveland dotplot [81] according to Zuur’s protocol [82] before correlation analyses. In total, 69 and 61 collections (*P. glauca* and *P. mariana*, respectively) were used in correlation analyses involving *T_b_* and *TH_50_*. All collections were used in correlation analyses for seed viability and final germination.

Correlation analysis was also conducted within each of the two major ecozones, the Boreal Plains and Boreal Shield, for each species. Correlations between final germination with climatic and geographic variables were analyzed for each germination temperature regime. GLM was used to determine seed viability as affected by ecozone and Duncan’s multiple-comparison test was used for mean separation. Significance level was assumed at *p* ≤ 0.05.

## 5. Conclusions

In conclusion, site temperature was the main factor controlling seed germination ability of the two spruce species. Seed viability was lower in northern seed collections and germination capacity was diminished at lower temperatures for both *P. glauca* and *P. mariana.* Overall, *P. mariana* showed better adaption to wet environments and was projected to initiate germination at higher temperature or later in the spring than *P. glauca*. Therefore, *P. mariana* may be better suited to warmer ambient temperatures than *P. glauca* under the same moisture conditions. Results from this study can be used to aid in modelling range shifts, migration patterns, or improve assumptions regarding the impacts of current and future climate changes. Climatic warming may affect the timing and success of seed germination and, thus, regeneration success in Canadian naturally regenerated boreal forests.

## Figures and Tables

**Table 1:** Correlation coefficients between seed viability and baseline climatic variables (1971–2000) across the Canadian boreal forest and within two selected ecozones for *Picea glauca* and *P. mariana*.

Variable		*P. glauca*		*P. mariana*	
		Boreal Plains	All Ecozones	Boreal Shield	All Ecozones
Start of growing season		0.36	0.16	−0.42 *	−0.15
End of growing season		−0.41 *	0.10	0.25	0.19
Length of growing season		−0.40 *	−0.01	0.40 *	0.22
Potential evaporation (PE)					
	Annual	0.42 *	−0.05	0.53 **	0.30 *
	Feb.	0.18	−0.25 *	0.31	0.05
	Apr.	0.00	−0.12	0.5 **	0.25 *
	May	0.39 *	−0.06	0.5 **	0.23
	Jun.	0.49 **	−0.08	0.35	0.19
	Jul.	0.45 *	−0.09	0.42 *	0.22
	Aug.	0.50 **	−0.07	0.43 *	0.22
	Sep.	0.40 *	0.03	0.59 **	0.36 **
	Oct.	0.27	0.14	0.57 **	0.34 **
Mean temperature					
	Annual	0.04	0.07	0.40 *	0.26 *
	Apr.	−0.30	−0.15	0.43 *	0.16
	May	0.12	−0.06	0.45 *	0.24
	Jul.	0.17	0.04	0.41 *	0.27 *
	Aug.	0.19	0.09	0.45 *	0.29 *
	Sep.	0.06	0.09	0.46 *	0.27 *
	Oct.	−0.05	0.12	0.47 **	0.28 *
	Nov.	0.19	0.18	0.34	0.25 *
Maximum temperature					
	Annual	0.14	0.04	0.51 **	0.34 **
	Feb.	0.01	0.01	0.35	0.25 *
	Mar.	0.00	−0.10	0.43 *	0.26 *
	Apr.	−0.20	−0.20	0.5 **	0.17
	May	0.25	−0.08	0.45 *	0.24
	Jul.	0.31	−0.02	0.45 *	0.32 **
	Aug.	0.36	0.03	0.49 **	0.34 **
	Sep.	0.25	0.05	0.51 **	0.32 **
	Oct.	0.14	0.11	0.52 **	0.32 **
	Nov.	0.19	0.18	0.38 *	0.27 *
Minimum temperature					
	Apr.	−0.40 *	−0.07	0.26	0.11
	May	−0.15	−0.01	0.40 *	0.21

* *p* ≤ 0.05, ** *p* ≤ 0.01. Ecozones and climatic variables not significantly correlated with seed viability are not listed.

**Table 2:** Correlation coefficients between final germination percentage, viability, and geographic variable across the Canadian boreal forest and within two selected ecozones.

Species	Ecozone	Geographic Variable	Germination Temperature °C						Viability
			12.5	15	17.5	20	25	30	
*P. glauca*	All ecozones	Longitude	−0.03	−0.03	0.00	0.12	0.15	0.35 **	0.24 *
		Latitude	0.18	0.18	0.16	0.02	−0.08	−0.31 *	−0.24 *
	Boreal Plains	Longitude	−0.10	0.15	0.30	0.30	0.21	−0.14	0.30
		Latitude	−0.29	−0.41	−0.37	−0.36	−0.33	−0.11	−0.43 *
	Boreal Shield	Longitude	0.63 **	0.73 **	0.79 **	0.66 **	0.45 *	0.49 *	0.10
		Latitude	−0.07	−0.11	−0.12	−0.18	−0.18	−0.29	0.05
*P. mariana*	All ecozones	Longitude	0.63 **	0.74 **	0.71 **	0.24	0.22	0.16	0.17
		Latitude	−0.42 **	−0.58 **	−0.67 **	−0.26 *	−0.24	−0.18	−0.33 **
	Boreal Plains	Longitude	−0.03	0.12	0.13	0.41	0.00	−0.26	0.08
		Latitude	−0.13	−0.20	−0.36	−0.27	0.01	0.37	−0.01
	Boreal Shield	Longitude	0.54 **	0.63 **	0.46 *	0.34	0.34	0.36	0.03
		Latitude	−0.10	−0.18	−0.25	−0.37	−0.39 *	−0.43 *	−0.55 **

* *p* ≤ 0.05, ** *p* ≤ 0.01.

**Table 3:** Correlation coefficients between seed germination traits (base temperature, *T_b_* and thermal time requirement for 50% germination, *TH_50_*) and geographic variables across the Canadian boreal forest and within two selected ecozones.

Species	Ecozone	Variables	*Tb* (°C)	*TH_50_* (d °C)
*P. glauca*	All ecozones	Longitude	0.01	0.16
	(n = 69)	Latitude	−0.00	−0.38 **
	Boreal Plains	Longitude	−0.06	−0.17
	(n = 23)	Latitude	0.17	0.23
	Boreal Shield	Longitude	−0.58 **	−0.52 **
	(n = 24)	Latitude	0.28	−0.05
*P. mariana*	All ecozones	Longitude	−0.22	−0.21
	(n = 61)	Latitude	0.16	0.17
	Boreal Plains	Longitude	0.56 **	−0.13
	(n = 20)	Latitude	−0.66 **	0.24
	Boreal Shield	Longitude	−0.18	−0.24
	(n = 26)	Latitude	0.19	−0.07

** *p* ≤ 0.01.

**Table 4:** Correlation coefficients between base temperature (*T_b_*) and baseline climatic variables (1971–2000) across the Canadian boreal forest and within two selected ecozones for *Picea glauca* and *P. mariana*.

Climate Variable		*P. glauca*		*P. mariana*	
		All Ecozones (n = 69)	Boreal Shield (n = 24)	All Ecozones (n = 61)	Boreal Plains (n = 20)
End of growing season		−0.09	−0.46 **	−0.15	−0.05
Potential evaporation (PE)					
	Annual	0.15	0.51 **	0.20	0.44 *
	May	0.22	0.50 **	0.17	0.34
	Jun.	0.22	0.57 **	0.18	0.42
	Jul.	0.23	0.62 **	0.25 *	0.52 **
	Aug.	0.19	0.63 **	0.29 *	0.49 **
	Sep.	0.10	0.36	0.13	0.50 **
	Nov.	−0.23	−0.54 **	−0.06	0.23
Precipitation (P)					
	Annual	−0.09	−0.69 **	−0.23	0.02
	CV	0.17	0.59 **	0.23	−0.07
	Jan.	−0.16	−0.64 **	−0.20	−0.28
	Feb.	−0.16	−0.61 **	−0.20	−0.27
	Mar.	−0.14	−0.66 **	−0.19	0.16
	Apr.	−0.11	−0.68 **	−0.21	0.24
	May	−0.04	−0.70 **	−0.23	0.21
	Jul.	0.22	−0.33	−0.32 *	−0.19
	Aug.	0.04	−0.66 **	−0.25	0.05
	Sep.	−0.01	−0.62 **	−0.24	0.28
	Oct.	−0.11	−0.65 **	−0.20	0.16
	Nov.	−0.13	−0.67 **	−0.21	−0.06
	Dec.	−0.19	−0.70 **	−0.20	−0.35
PE-P		−0.12	−0.69 **	−0.25 *	−0.22
Temperature seasonality (CV)		0.23	0.65 **	0.14	0.27
Temperature annual range		0.27 *	0.64 **	0.12	0.25
Mean temperature					
	Annual	−0.13	−0.49 **	−0.07	0.28
	Jan.	−0.19	−0.59 **	−0.11	−0.09
	Feb.	−0.16	−0.51 **	−0.05	−0.10
	May	0.15	0.44 **	0.11	0.39
	Jun.	0.18	0.50 **	0.11	0.43
	Jul.	0.16	0.51 **	0.10	0.46 **
	Aug.	0.12	0.42 *	0.07	0.49 **
	Nov.	−0.10	−0.58 **	−0.16	0.40
	Dec.	−0.18	−0.61 **	−0.12	0.04
Maximum temperature					
	Jan.	−0.16	−0.58 **	−0.14	−0.11
	Feb.	−0.12	−0.44 **	−0.09	−0.21
	Apr.	0.09	0.42 *	0.09	−0.19
	May	0.2	0.51 **	0.12	0.33
	Jun.	0.23	0.55 **	0.12	0.35
	Jul.	0.22	0.6 **	0.14	0.38
	Aug.	0.20	0.58 **	0.15	0.41
	Nov.	−0.10	−0.54 **	−0.16	0.27
	Dec.	−0.17	−0.59 **	−0.15	−0.04
Minimum temperature					
	Jan.	−0.21	−0.59 **	−0.08	−0.06
	Feb.	−0.19	−0.53 **	−0.02	0.01
	Mar.	−0.13	−0.48 **	−0.01	0.13
	Jun.	0.11	0.34	0.07	0.47 **
	Jul.	0.07	0.23	0.03	0.49 **
	Aug.	0.02	−0.03	−0.02	0.49 **
	Oct.	−0.08	−0.47 **	−0.16	0.09
	Nov.	−0.11	−0.61 **	−0.15	0.47 **
	Dec.	−0.19	−0.61 **	−0.10	0.11

* *p* ≤ 0.05, ** *p* ≤ 0.01.

**Table 5:** Correlation coefficients between *TH_50_* and baseline climatic variables (1971–2000) across the Canadian boreal forest and within two selected ecozones for *Picea glauca*. No significant correlation was found in *P. mariana*.

Variable		All Ecozones (n = 69)	Boreal Plains (n = 23)	Boreal Shield (n = 24)
Start of growing season		−0.38 **	−0.32	−0.25
End of growing season		0.34 **	0.37	−0.25
Length of growing season		0.42 **	0.36	−0.02
Potential evaporation (PE)				
	Annual	0.36 **	−0.21	0.45 *
	Feb.	0.32 **	0.02	−0.10
	Mar.	0.44 **	−0.01	−0.24
	Apr.	0.39 **	0.03	0.30
	May	0.19	−0.18	0.47 *
	Jun.	0.19	−0.27	0.48 *
	Jul.	0.28 *	−0.26	0.49 *
	Aug.	0.22	−0.32	0.49 *
	Sep.	0.37 **	−0.19	0.36
	Oct.	0.42 **	−0.10	0.13
	Nov.	0.36 **	−0.11	−0.35
Precipitation (P)				
	Annual	0.23	0.36	−0.41 *
	CV	−0.33 **	0.25	0.40
	Jan.	0.24 *	0.35	−0.35
	Feb.	0.23	0.43 *	−0.41 *
	Mar.	0.25 *	0.10	−0.41 *
	Apr.	0.23	−0.33	−0.47 *
	May	0.28 *	0.11	−0.37
	Jul.	0.03	0.44 *	−0.40
	Aug.	0.11	0.40 *	−0.41 *
	Nov.	0.24 *	0.14	−0.39
PE -P		0.13	0.46 *	−0.46 *
Temperature annual range		−0.15	−0.11	0.42 *
Mean temperature				
	Annual	0.44 **	0.04	−0.10
	Jan.	0.31 *	0.02	−0.32
	Feb.	0.34 **	0.03	−0.23
	Mar.	0.40 **	0.04	−0.15
	Apr.	0.38 **	0.26	0.23
	May	0.36 **	−0.04	0.41 *
	Jun.	0.38 **	−0.08	0.41 *
	Jul.	0.45 **	−0.09	0.40
	Aug.	0.46 **	−0.11	0.33
	Sep.	0.47 **	0.06	0.03
	Oct.	0.40 **	0.19	−0.09
	Nov.	0.35 **	−0.06	−0.34
	Dec.	0.32 **	0.03	−0.34
Maximum temperature				
	Annual	0.46 **	−0.02	0.14
	Jan.	0.32 **	0.02	−0.28
	Feb.	0.36 **	0.03	−0.11
	Mar.	0.43 **	0.05	0.11
	Apr.	0.34 **	0.21	0.45 *
	May	0.29 *	−0.16	0.47 *
	Jun.	0.33 **	−0.23	0.46 *
	Jul.	0.43 **	−0.24	0.45 *
	Aug.	0.43 **	−0.26	0.42 *
	Sep.	0.46 **	−0.11	0.23
	Oct.	0.39 **	0.05	0.08
	Nov.	0.36 **	−0.05	−0.3
	Dec.	0.33 **	0.03	−0.31
Minimum temperature				
	Annual	0.39 **	0.14	−0.27
	Jan.	0.29 *	0.03	−0.35
	Feb.	0.32 **	0.03	−0.30
	Mar.	0.36 **	0.02	−0.27
	Apr.	0.36 **	0.31	−0.18
	May	0.41 **	0.18	0.21
	Jun.	0.39 **	0.12	0.28
	Jul.	0.39 **	0.07	0.19
	Aug.	0.39 **	0.10	0.03
	Sep.	0.39 **	0.27	−0.22
	Oct.	0.37 **	0.34	−0.25
	Nov.	0.34 **	−0.09	−0.37
	Dec.	0.32 **	0.04	−0.36

* *p* ≤ 0.05, ** *p* ≤ 0.01.

## Data Availability

The original contributions presented in this study are included in the article/Supplementary Materials. Further inquiries can be directed to the corresponding author.

## Supplementary Materials

The following supporting information can be downloaded at https://www.mdpi.com/article/10.3390/plants15060882/s1. Table S1: Basic information on seed sources.

## Abbreviations

The following abbreviations are used in this manuscript:

PE	Potential Evaporation
SDM	Species Distribution Modelling
*T_b_*	Base Temperature
*TH_50_*	Thermal Time Requirement for 50% Germination
*T_o_*	Optimal Temperature
