# Supplementary material for: Spatial Variations in Seed Germination Traits of White Spruce (Picea glauca) and Black Spruce (P. mariana) Across the Canadian Boreal Forest
Source: Plants (Basel). 2026 Mar 12;15(6):882. doi: 10.3390/plants15060882 (PMC13029439; doi:10.3390/plants15060882)
Supplement: Supplementary file 1 [file plants-15-00882-s001.zip › plants-4167049-supplementary.pdf]

Table S1. Basic information of seed sources

Species	Collections	Ecozones	Longitude	Latitude	1000-seed Weight (g)	Viability (%)
Picea glauca	1	Arctic Cordillera	-65.4	45.4	2.1	94.0
	2		-65.2	46.7	2.5	100.0
	3		-65.3	43.8	2.3	90.0
	4		-61.5	45.2	2.4	100.0
	5		-60.8	46.0	1.9	82.0
	6		-60.8	46.0	-	88.0
	7		-70.4	46.0	2.8	86.0
	8		-69.1	47.5	2.6	86.0
	9		-64.8	48.3	2.7	92.0
	10	Boreal Cordillera	-136.4	63.4	2.1	78.0
	11		-128.9	60.1	2.4	63.5
	12	Boreal Plains	-118.1	56.4	2.2	98.0
	13		-115.3	52.6	2.6	98.0
	14		-114.8	51.6	1.7	88.0
	15		-114.6	56.6	2.1	72.0
	16		-112.6	56.2	2.4	95.0
	17		-101.8	54.5	2.1	94.0
	18		-100.7	51.7	2.3	94.0
	19		-97.6	51.0	2.0	82.0
	20		-108.5	55.9	2.1	54.4
	21		-108.4	54.0	2.0	98.0
	22		-101.8	52.7	2.2	98.0
	23		-112.6	56.2	2.1	92.0
	24		-105.9	54.2	-	98.0
	25		-102.9	52.9	-	84.0
	26		-107.9	53.9	-	88.0
	27		-102.1	53.5	-	97.0
	28		-107.0	54.5	-	89.0
	29		-105.8	54.5	-	94.0
	30		-107.0	54.7	-	97.0
	31		-102.3	53.2	-	92.0
	32		-102.5	53.0	-	99.0
	33		-102.4	52.3	-	98.0
	34		-105.6	53.4	-	98.0
	35		-102.6	52.9	-	86.0
	36	Boreal Shield	-98.0	55.5	1.8	98.0
	37		-96.1	49.7	2.6	87.6
	38		-60.2	53.3	2.1	94.0
	39		-59.1	48.0	2.9	84.0
	40		-58.3	49.3	2.9	100.0
	41		-55.4	51.4	2.5	94.0
	42		-54.0	48.2	2.9	66.5
	43		-54.0	48.2	2.9	94.0
	44		-93.9	49.0	1.6	100.0
	45		-93.8	51.0	2.4	88.0

Table S1. Continued

Species	Collections	Ecozones	Longitude	Latitude	1000-seed Weight (g)	Viability (%)
Picea glauca	46	Boreal Shield	-89.9	48.6	2.6	86.0
	47		-89.1	50.2	2.2	94.0
	48		-89.1	49.5	1.8	89.6
	49		-84.6	49.6	2.2	74.0
	50		-83.6	47.3	2.2	90.0
	51		-81.6	48.6	2.2	100.0
	52		-75.3	46.8	2.2	94.0
	53		-75.1	45.9	2.5	98.0
	54		-74.9	48.7	2.3	88.0
	55		-68.6	49.2	1.8	96.0
	56		-64.1	49.9	2.5	92.0
	57		-93.3	50.2	2.4	54.0
	58		-94.1	49.5	2.2	83.8
	59	Hudson Plains	-94.1	58.8	2.1	98.0
	60	Mixedwood Plains	-82.0	45.7	2.0	96.5
	61		-77.3	44.2	2.7	92.0
	62		-77.1	44.2	2.7	98.0
	63		-75.6	44.9	2.4	86.0
	64		-77.3	44.2	2.4	98.0
	65		-82.0	45.7	2.0	98.0
	66	Montane Cordillera	-125.0	52.8	2.3	96.0
	67		-118.9	49.3	2.4	63.3
	68	Prairies	-109.9	49.5	3.1	86.0
	69	Taiga Plains	-115.0	58.6	2.1	92.0
Picea mariana	70	Arctic Cordillera	-75.4	47.2	1.1	100.0
	71		-67.3	46.8	1.5	100.0
	72		-65.4	45.8	1.7	100.0
	73		-62.2	45.0	1.4	96.0
	74		-60.1	46.0	1.4	94.0
	75		-64.0	46.5	1.6	100.0
	76		-70.9	45.6	1.6	87.3
	77		-70.0	46.9	1.4	100.0
	78		-68.0	48.1	1.6	100.0
	79		-64.5	48.5	1.5	85.3
	80	Boreal Cordillera	-61.9	47.3	1.5	90.0
	81		-136.4	63.2	1.2	82.0
	82	Boreal Plains	-133.9	60.3	1.0	90.0
	83		-116.2	54.7	1.2	100.0
	84		-116.0	56.0	1.3	75.6
	85		-111.8	56.9	1.0	94.0
	86		-100.7	51.6	1.0	92.0
	87		-96.4	50.7	0.9	84.0
	88		-106.2	53.2	1.1	82.0
	89		-102.7	53.1	1.0	92.0

Table S1. Continued

Species	Collections	Ecozones	Longitude	Latitude	1000-seed Weight (g)	Viability (%)
Picea mariana	90	Boreal Plains	-105.6	54.8	-	98.0
	91		-102.5	52.3	-	95.0
	92		-105.9	54.4	-	100.0
	93		-105.0	54.1	-	94.0
	94		-105.9	54.7	-	93.0
	95		-106.9	54.8	-	91.0
	96		-106.7	54.5	-	100.0
	97		-107.6	53.8	-	99.0
	98		-108.1	52.2	-	98.0
	99		-103.2	52.9	-	99.0
	100		-104.5	53.8	-	86.0
	101		-102.1	53.5	-	100.0
	102	Boreal Shield	-97.7	55.6	0.8	88.0
	103		-60.5	53.5	1.2	95.6
	104		-57.3	49.3	1.6	83.5
	105		-56.0	49.5	1.4	100.0
	106		-54.4	47.9	1.5	100.0
	107		-53.9	49.1	1.4	94.0
	108		-94.4	49.8	1.1	98.0
	109		-91.3	53.0	1.0	100.0
	110		-86.6	49.5	0.8	94.0
	111		-84.2	49.5	0.9	100.0
	112		-83.2	47.9	0.9	100.0
	113		-81.5	47.5	1.0	100.0
	114		-80.5	49.3	0.9	100.0
	115		-77.3	45.8	1.5	100.0
	116		-79.0	47.4	1.1	100.0
	117		-77.9	47.6	1.1	100.0
	118		-77.2	50.1	1.0	96.0
	119		-74.6	46.1	1.4	98.0
	120		-74.1	48.4	1.1	98.0
	121		-74.0	50.7	1.1	91.8
	122		-74.0	50.7	1.2	92.0
	123		-72.0	49.2	1.1	91.8
	124		-70.9	50.3	0.9	82.0
	125		-68.5	49.7	1.1	86.0
	126		-66.0	50.3	1.4	86.0
	127		-72.7	45.4	1.3	96.0
	128	Mixedwood Plains	-72.6	46.7	1.5	90.0
	129		-105.4	55.1	1.0	96.0
	130	Taiga Plains	-105.6	54.8	-	98.0

Due to the limited number of seeds for collection 6, 24~35 and 90~101, the 1000-seed weight was not measured.
